# Accelerometer-determined physical activity and self-reported health in a population of older adults (65–85 years): a cross-sectional study

**DOI:** 10.1186/1471-2458-14-284

**Published:** 2014-03-27

**Authors:** Hilde Lohne-Seiler, Bjorge H Hansen, Elin Kolle, Sigmund A Anderssen

**Affiliations:** 1 Norwegian School of Sport Sciences, Department of Sport Medicine, P.B. 4014 Ullevaal Stadion, 0806 Oslo, Norway; 2 University of Agder, Faculty of Health and Sport Sciences, Service Box 422, 4604 Kristiansand, Norway

**Keywords:** Physical activity level, Self-reported health, Accelerometer, Older people

## Abstract

**Background:**

The link between physical activity (PA) and prevention of disease, maintenance of independence, and improved quality of life in older adults is supported by strong evidence. However, there is a lack of data on population levels in this regard, where PA level has been measured objectively. The main aims were therefore to assess the level of accelerometer-determined PA and to examine its associations with self-reported health in a population of Norwegian older adults (65–85 years).

**Methods:**

This was a part of a national multicenter study. Participants for the initial study were randomly selected from the national population registry, and the current study included those of the initial sample aged 65–85 years. The ActiGraph GT1M accelerometer was used to measure PA for seven consecutive days. A questionnaire was used to register self-reported health. Univariate analysis of variance with Bonferroni adjustments were used for comparisons between multiple groups.

**Results:**

A total of 560 participants had valid activity registrations. Mean age (SD) was 71.8 (5.6) years for women (n = 282) and 71.7 (5.2) years for men (n = 278). Overall PA level (cpm) differed considerably between the age groups where the oldest (80–85 y) displayed a 50% lower activity level compared to the youngest (65–70 y). No sex differences were observed in overall PA within each age group. Significantly more men spent time being sedentary (65–69 and 70–74 years) and achieved more minutes of moderate to vigorous PA (MVPA) (75–79 years) compared to women. Significantly more women (except for the oldest), spent more minutes of low-intensity PA compared to men. PA differed across levels of self-reported health and a 51% higher overall PA level was registered in those, with “very good health” compared to those with “poor/very poor health”.

**Conclusion:**

Norwegian older adults PA levels differed by age. Overall, the elderly spent 66% of their time being sedentary and only 3% in MVPA. Twenty one percent of the participants fulfilled the current Norwegian PA recommendations. Overall PA levels were associated with self-reported health.

## Background

Regular physical activity in older adults is critically important for healthy aging [1]. The link between regular physical activity and disease prevention, maintenance of independence and improved quality of life is supported by strong evidence [2,3]. However, there is a lack of knowledge on the physical activity levels and sedentary behavior among older people. Current knowledge is primarily based on studies using subjective assessment methods (e.g. questionnaires). Recalling physical activity is a complex cognitive task, and old adults are likely to have particular memory and recall skill limitations [4-6].

The introduction of accelerometers for objective assessment of physical activity allows for valid and reliable assessments of activity intensity, frequency, and duration [7,8]. Accelerometry is less prone to the recall and social desirability biases associated with self-report instruments [9]. Objective information on the physical activity levels and sedentary behavior has the potential to increase our understanding of physical activity in old age [3].

There are only a limited number of studies that have assessed physical activity using accelerometers in older adults. Most of these studies were completed in the USA [10-12], Canada [13] and the United Kingdom [14,15] and relatively few studies are anchored in the northern European countries [16-18]. Additionally, there is a lack of knowledge regarding physical activity levels in adults over 79 years of age [11,13,18].

The World Health Organization recommends that information on how individuals perceive their own health should be collected in population-based studies including older individuals [19]. Self-reported health status is considered as a sensitive measure of overall health in older adults, influenced by physical function, the presence of disease, the existence of disabilities, functional limitations, and the rate of aging [20]. It is viewed as a holistic measurement of health, reflecting both physical and mental health as well as well-being [21]. At present, few studies have examined physical activity level measured objectively in the elderly in combination with self-report instruments including simple measures of health [22].

The aims of the present study were therefore to describe the level of accelerometer-determined physical activity in a random national sample of Norwegian older adults (65–85 years), and secondary to investigate the associations between physical activity level and self-reported health.

## Methods

### Design

This study was part of a national multicenter study involving 10 test centers throughout Norway [23]. The sample included in this study is those aged 65 to 85 years (categorized into the age groups 65–69 years, 70–74 years, 75–79 years, and 80–85 years). From the Norwegian population registry a representative sample of 2040 individuals aged 65–85 years were drawn from the geographical areas surrounding the involved test centers, and study information and informed consent were distributed via mail to the drawn sample. Written informed consent was obtained from 628 subjects (313 women and 315 men, a total of 31% of the invited sample). Those with valid accelerometer data (accumulated at least 10 hours of valid activity recordings per day for at least four days) were included in the final data analysis (n = 560, 282 women and 278 men). The study was approved by the Regional Committee for Medical and Health Research Ethics and the Norwegian Social Science Data Services AS.

### Measurement of physical activity

We used ActiGraph GT1M accelerometers (ActiGraph, LLC, Pensacola, FL) to measure the participants’ physical activity levels [23]. The accelerometer registers vertical acceleration in units called counts, and collects data at a rate of 30 times per second in user-defined sampling intervals (epochs). The number of steps taken per day was registered using the embedded pedometer function. The participants received a pre-programmed accelerometer by mail. They were instructed to wear the accelerometer over the right hip in an elastic band while awake, and to remove the accelerometer when doing water activities. The participants wore the accelerometer for seven consecutive days, and they returned the accelerometer by prepaid express mail after the registration period.

We initialized and downloaded the accelerometers using ActiLife software provided by the manufacturer (ActiGraph LLC, Pensacola, FL). Customized SAS based macros (SAS Institute Inc., Cary, NC, USA) were used to reduce the data and derive the following variables: 1) mean counts per minute (cpm); 2) number of steps taken per day (spd); 3) number of minutes spent in intensity-specific categories, and 4) percentage of the study population meeting the national PA recommendations (minimum of 30 minutes of daily moderate PA in bouts of 10 minutes or more) [24]. The following intensity-specific cut-points were applied to the raw data; sedentary time was defined as all activity below 100 cpm (e.g. sitting, reclining, lying down) [25,26], low-intensity PA was defined as all activity between 100 and 759 cpm (e.g. washing dishes, hanging washing, ironing, cooking, eating, working at a computer desk or performing other office duties) [18], and time in lifestyle activity (e.g. slow walking, grocery shopping, vacuuming, child care) was defined as all activity between 760 and 2019 cpm [18,27]. Moderate-to-vigorous PA (MVPA) was defined as all activity ≥2020 cpm (e.g. walking at speeds of ≥78 m · min^−1^ or more vigorous activities) [12]. The number of minutes per day at different intensities was determined by summing all minutes where the count met the criterion for the specific intensity, divided by the number of valid days.

Activity files were deemed valid if a participant accumulated at least 10 hours of valid activity recordings per day for at least four days, which is in accordance with the suggestions by Trost, McIver, and Pate [28]. Wear time was defined by subtracting non-wear time from 18 hours (all data between 00:00 and 06:00 were excluded). Non-wear time was defined as intervals of at least 60 consecutive minutes with zero counts, with allowance for 1 minute with counts greater than zero.

### Other variables

The participants self-reported data on anthropometry (body height and body mass), level of education level and perceived health through a questionnaire. Body mass index (BMI) was computed as body mass (kg) divided by height in meters squared (m^2^). Level of education was categorized into four groups: less than high school, high school, less than four years of university education, and university education for four years or more. Perceived health was reported as “very good health”, “good health”, “either good or bad health”, and “poor/very poor health”. Self-reported perceived health scale was condensed from five to four categories. “Very good health”, “good health” and “either good or bad health” were kept in separate categories, while “poor health” and “very poor health” were combined into one category “poor/very poor health”. This was due to the low numbers in the “poor” and “very poor health” groups.

In addition, the participants also recorded if they were retired or in part-time/full-time employment.

### Statistical analysis

All statistical analyses were conducted using IBM SPSS Statistics 19 for Windows (IBM Corporation, Route, Somers, NY, USA).

We assessed differences in continuous variables (age, height, body mass, BMI, number of minutes spent in intensity-specific categories) between women and men in the different age groups using Student’s t-test for independent samples. We used Pearson’s chi-square analyses to identity differences between the sexes in education level and self-reported health, and in the proportion of participants from each sex who adhered to the current PA recommendations.

Univariate analysis of variance with Bonferroni adjustments were used for comparisons between multiple groups. Overall physical activity level (cpm and spd) varied between test centers and with age, and these variables were therefore treated as potential confounders. When studying the differences in PA measurements (both cpm and time in different intensity categories) by age and sex the analysis were adjusted for test center (Tables 1 and 2).

**Table 1:** Physical activity measurements by age and sex

	**Women**		**Men**		**Mean difference**	**95% CI**	**All**	
**Age**	**N**	**Mean**	**N**	**Mean**	**(Men-Women)**		**N**	**Mean**
**Overall PA (cpm)**^**a, b**^								
65–69 yr	127	311 (13.4)	116	325 (14.0)	14 (19.6)	−25 to 52	243	317 (9.2)^c^
70–74 yr	67	294 (19.2)	79	308 (17.7)	14 (26.1)	−38 to 65	146	301 (11.8)^d^
75–79 yr	51	215 (19.5)	55	256 (18.8)	41 (27.1)	−13 to 95	106	237 (13.9)^e^
80–85 yr	37	166 (11.2)	28	153 (12.8)	−13 (17.1)	−47 to 21	65	160 (17.7)^f^
**Steps per day**^**a, b**^								
65–69 yr	127	7537 (1825.1)	116	11191 (1886.5)	3654 (2646.5)	−1559 to 8867	243	9302 (866.1)^g^
70–74 yr	67	6904 (387.6)	79	6798 (353.0)	−106 (524.3)	−1143 to 930	146	6841 (1109.1)
75–79 yr	51	5256 (433.7)	55	6114 (417.9)	859 (602.8)	−336 to 2054	106	5721 (1307.5)
80–85 yr	37	4059 (305,9)	28	3436 (348.8)	−623 (464.3)	−1550 to 304	65	3777 (1635.4)^h^

^*a*^*Data are presented as mean standard error of the mean (SEM).*

^*b*^*All values (overall PA in cpm and in steps per day) are adjusted for test centre.*

^*c*^*65–69 yr compared to 75–79 yr p = 0.000, and 65–69 yr compared to 80–85 yr p = 0.000.*

^*d*^*70–74 yr compared to 75–79 yr p = 0.03, and 70–74 yr compared to 80–85 yr p = 0.000.*

^*e*^*75–79 yr compared to 65–69 yr p = 0.000, 75–79 yr compared to 70–74 yr p = 0.03, and 75–79 yr compared to 80–85 yr p = 0.04.*

^*f*^*80–85 yr compared to 65–69 yr p = 0.000, 80–85 yr compared to 70–74 yr p = 0.000, and 80–85 yr compared to 75–79 yr p = 0.04.*

^*g*^*65–69 yr compared to 80–85 yr p = 0.02.*

^*h*^*80–85 yr compared to 65-79 yr p=0.02.*

No significant differences between sex within age groups.

**Table 2:** **Mean ± SEM minutes per day**^**a **^**of sedentary activity, low PA, lifestyle PA, and MVPA**

	**Women (n = 282)**		**Men (n = 278)**		**Mean difference**	**95% CI**	**All (n = 560)**	
**Age**	**N**	**Mean ± SEM**	**N**	**Mean ± SEM**	**(Women-Men)**		**N**	**Mean ± SEM**
**Sedentary PA**								
65–69 yr	127	535 (6.9)^b^	116	558 (7.3)	−23.1*	−42.9 to −3.3	243	547 (5.0)^e^
70–74 yr	67	525 (9.5)^c^	79	554 (8.7)	−28.9*	−54.4 to −3.5	146	541 (6.4)^f^
75–79 yr	51	561 (12.1)	55	580 (10.1)	−18.3	−49.6 to 13.0	106	571 (7.6)^g^
80–85 yr	37	592 (12.5)^d^	28	590 (11.5)	1.6	−32.3 to 35.6	65	591 (9.4)^h^
**Low-intensity PA**								
65–69 yr	127	223 (4.9)^i^	116	192 (4.4)^m^	30.9*	17.9 to 43.7	243	208 (3.5)^p^
70–74 yr	67	223 (6.4)^j^	79	187 (5.6)^n^	36.5*	19.7 to 53.3	146	203 (4.4)^q^
75–79 yr	51	200 (7.5)^k^	55	179 (7.6)	20.4*	−0.3 to 41.1	106	189 (5.2)^r^
80–85 yr	37	178 (8.6)^l^	28	157 (9.9°)	21.4	−4.7 to 47.5	65	169 (6.5)^s^
**Lifestyle PA**								
65–69 yr	127	69 (3.2)^t^	116	67 (3.8)^x^	1.4	−8.4 to 11.2	243	68 (2.3)^bb^
70–74 yr	67	64 (5.0)^u^	79	65 (4.3)^y^	−1.6	−14.6 to 11.4	146	65 (3.0)^cc^
75–79 yr	51	49 (5.4)^v^	55	54 (4.9)^z^	−5.3	−19.7 to 9.1	106	52 (3.5)^dd^
80–85 yr	37	37 (3.6)^w^	28	31 (3.5)^aa^	5.3	−4.6 to 15.7	65	34 (4.3)^ee^
**MVPA**								
65–69 yr	127	32 (2.2)^ff^	116	36 (2.5)^jj^	−4.8	−11.4 to 1.9	243	34 (1.6)^nn^
70–74 yr	67	28 (3.0)^gg^	79	31 (2.9)^kk^	−2.6	−10.9 to 5.7	146	29 (2.0)^oo^
75–79 yr	51	17 (2.4)^hh^	55	27 (3.8)^ll^	−9.9*	−18.9 to −0.9	106	22 (2.4)^pp^
80–85 yr	37	10 (2.1)^ii^	28	9.0 (1.5)^mm^	1.3	−3.8 to 6.4	65	9 (2.9)^qq^

*p ≤ 0.05 for sex within age group.

^*a*^*All values (mean ± SEM minutes per day of sedentary activity, low PA, lifestyle PA, and MVPA) are adjusted for test centre.*

^*b*^*65–69 yr compared to 80–85 yr p = 0.001.*

^*c*^*70–74 yr compared to 80–85 yr p = 0.000.*

^*d*^*80–85 yr compared to 65–69 yr p = 0.001, 80–85 yr compared to 70–74 yr p = 0.000.*

^*e*^*65–69 yr compared to 75–79 yr p = 0.05, 65–69 yr compared to 80–85 yr p = 0.000.*

^*f*^*70–74 yr compared to75–79 yr p = 0.02, 70–74 yr compared to 80–85 yr p = 0.000.*

^*g*^*75–79 yr compared to 65–69 yr p = 0.05, 75–79 yr compared to 70–74 yr p = 0.02.*

^*h*^*80–85 yr compared to 65–69 yr p = 0.000, 80–85 yr compared to 70–74 yr p = 0.000.*

^*1*^*65–59 yr compared to 75–79 yr p = 0.05, 65–69 yr compared to 80–85 yr p = 0.000.*

^*j*^*70–74 yr compared to 80–85 yr p = 0.000.*

^*k*^*75–79 yr compared to 65–69 yr p = 0.05.*

^*l*^*80–85 yr compared to 65–69 yr p = 0.000, 80–85 yr compared to 70–74 yr p = 0.000.*

^*m*^*65–69 yr compared to 80–85 yr p = 0.006.*

^*n*^*70–74 yr compared to 80–85 yr p = 0.04.*

^*o*^*80–85 yr compared to 65–69 yr p = 0.006, 80–85 yr compared to 70–74 yr p = 0.04.*

^*p*^*65–69 yr compared to 75–79 yr p = 0.02, 65–69 yr compared to 80–85 yr p = 0.000.*

^*q*^*70–74 yr compared to 80–85 yr p = 0.000.*

^*r*^*75–79 yr compared to 65–69 yr p = 0.02.*

^*s*^*80–85 yr compared to 65–69 yr p = 0.000, 80–85 yr compared to 70–75 yr p = 0.000.*

^*t*^*65–69 yr compared to 75–79 yr p = 0.005, 65–69 yr compared to 80–85 yr p = 0.000.*

^*u*^*70–74 yr compared to 80–85 yr p = 0.001.*

^*v*^*75–79 yr compared to 65–69 yr p = 0.005.*

^*w*^*80–85 yr compared to 65–69 yr p = 0.000, 80–85 yr compared to 70–74 y p = 0.001.*

^*x*^*65–69 yr compared to 80–85 yr p = 0.000.*

^*y*^*70–74 yr compared to 80–85 yr p = 0.000.*

^*z*^*75–79 yr compared to 80–85 yr p = 0.04.*

^*aa*^*80–85 yr compared to 65–69 yr p = 0.000, 80–85 yr compared to 70–74 yr p = 0.000, 80–85 yr compared to 75–79 yr p = 0.04.*

^*bb*^*65–69 yr compared to 75–79 yr p = 0.001, 65–69 yr compared to 80–85 yr p = 0.000.*

^*cc*^*70–74 yr compared to 75–79 yr p = 0.04, 70–74 yr compared to 80–85 yr p = 0.000.*

^*dd*^*75–79 yr compared to 65–69 yr p = 0.001, 75–79 yr compared to 70–74 yr p = 0.04, 75–79 yr compared to 80–85 yr p = 0.008.*

^*ee*^*80–85 yr compared to 65–69 yr p = 0.000, 80–85 yr compared to 70–74 yr p = 0.000, 80–85 yr compared to 75–79 yr p = 0.008.*

^*ff*^*65–69 yr compared to 75–79 yr p = 0.001, 65–69 yr compared to 80–85 yr p = 0.000.*

^*gg*^*70–74 yr compared to 75–79 yr p = 0.05, 70–74 yr compared to 80–85 yr p = 0.001.*

^*hh*^*75–79 yr compared to 65–69 yr p = 0.001, 75–79 yr compared to 70–74 yr p = 0.05.*

^*ii*^*80–85 yr compared to 65–69 yr p = 0.000, 80–85 yr compared to 70–74 yr p = 0.001.*

^*jj*^*65–69 yr compared to 80–85 yr p = 0.000.*

^*kk*^*70–74 yr compared to 80–85 yr p = 0.001.*

^*ll*^*75–79 yr compared to 80–85 yr p = 0.01.*

^*mm*^*80–85 yr compared to 65–69 yr p = 0.000, 80–85 yr compared to 70–74 yr p = 0.001, 80–85 yr compared to 75–79 yr p = 0.01.*

^*nn*^*65–69 yr compared to 75–79 yr p = 0.000, 65–69 yr compared to 80–85 yr p = 0.000.*

^*oo*^*70–74 yr compared to 80–85 yr p = 0.000.*

^*pp*^*75–79 yr compared to 65–69 yr p = 0.000, 75–79 yr compared to 80–85 yr p = 0.004.*

^*qq*^*80–85 yr compared to 65–69 yr p = 0.000, 80–85 yr compared to 70–74 yr p = 0.000, 80–85 yr compared to 75–79 yr p = 0.004.*

**Figure 1:**
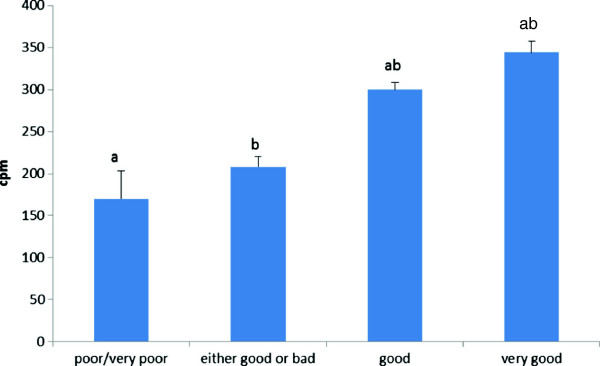
**Mean (SEM) overall PA levels in counts per minute (cpm) in the different self-reported health groups (“poor/very poor health”, “either good or bad health”, “good health”, and “very good health”).***a-b: Equal letter indicate significant difference (p<0.05) in overall PA level between the different self-reported health groups. All values are adjusted for age, BMI, education level, and test centre.*

Furthermore, BMI and education level varied across the categories of self-reported health, and thus treated as potential confounders. When examining the differences in overall PA levels in the different self-reported health groups, analysis were adjusted for test center, age, BMI, and education level (Figure 1). Linear regression analysis was used to estimate changes in physical activity level with increasing age.

## Results

### Physical characteristics of the study sample

Table 3 shows anthropometrical data, level of education and self-reported health of the study sample. The mean age (standard deviation (SD)) was 71.8 (5.6) years for women (n = 282) and 71.7 (5.2) years for men (n = 278). Overall, 34% of the participants reported an education level less than high school, 36% reported completing high school, and 30% reported to have a university education. The majority of the study sample reported having “very good health” (22.3% of women and 16.3% of men) or” good health” (56.2% of women and 53.7% of men). The majority (82%) of participants were retired whilst 11% were part time or full time employed. The remaining 6% didn’t report their occupation. In the youngest age group (65–69 years) 73% were retired (4% didn`t report their occupation) compared to 96% in the oldest age group (80–85 years) (p < 0.01).

**Table 3:** Physical characteristics, education level, and self-reported health of the study sample (n = 560) by age and sex

	**65–69 yr**		**70–74 yr**		**75–79 yr**		**80–85 yr**		**All**	
**Variable**	**Women**	**Men**	**Women**	**Men**	**Women**	**Men**	**Women**	**Men**	**Women**	**Men**
N	127	116	67	79	51	55	37	28	282	278
Age (yr)^a^									71.8 (5.6)	71.7 (5.2)
Height (cm)^a^	164.1 (5.4)	178.1 (5.9)*	163.4 (5.1)	177.1 (6.8)*	163.3 (5.0)	175.9 (8.5)*	163.8 (6.3)	175.4 (5.0)*	163.8 (5.4)	177.1 (6.7)*
Body mass (kg)^a^	67.8 (10.5)	84.7 (11.5)*	65.5 (10.4)	80.0 (11.9)*	63.4 (7.5)	77.2 (11.2)*	67.4 (11.1)	76.1 (10.5)*	66.4 (10.2)	81.0 (11.9)*
BMI (kg/m^2^)^a^	25.1 (3.7)	26.7 (3.0)*	24.5 (3.9)	25.4 (3.2)	23.8 (2.6)	25.0 (3.2)*	25.1 (3.5)	24.7 (2.9)	24.7 (3.6)	25.8 (3.2)*
Education level (%)										
Less than high school	38.8	28.1	37.3	38.0	42.0	25.9	26.8	38.7	37.3	31.6
High school	35.7	35.5	41.8	31.6	32.0	40.7	34.1	38.7	36.2	35.8
University <4 yr	10.9	20.7	11.9	20.3	20.0	16.7	24.4	9.7	14.6	18.6
University ≥4 yr	14.7	15.7	9.0	10.1	6.0	16.7	14.6	12.9	11.8	14.0
Self-reported health (%)										
Very good	22.3	16.3	20.9	23.5	9.8	10.9	14.3	18.8	18.6	17.5
Good	56.2	53.7	56.7	49.4	62.7	54.5	45.2	40.6	55.9	51.2
Either good or bad	19.2	27.6	19.4	27.2	23.5	27.3	31.0	31.3	21.7	27.8
Poor/very poor	2.3	2.4	3.0	0.0	3.9	7.3	9.5	9.4	3.8	3.4

^*a*^*Data are presented as mean (SD).*

*p < 0.05 between sex within age group and all.

### Physical activity measurements

A total of 560 participants had valid accelerometer data and were included in the analyses. There were no differences in anthropometrical data or level of education when comparing the participants who were included and those who were excluded (due to insufficient accelerometer wear time) from the final analysis. The participants achieved a mean of 6.6 days (SD 1.4) with valid activity recordings, and the mean wear time was 14.0 hours per day (SD 1.2). The PA variables (overall PA in cpm and steps per day across age and sex) are presented in Table 1.

#### Overall PA level across age

Overall physical activity level (cpm) was significantly different between the age groups, except between the age groups 65–69 and 70–74 years. This accounted for an overall PA level difference of 21% (p = 0.003) between the70-74 and 75–79 years age groups, and a 32% (p = 0.004) difference between the 75–79 and 80–85 years age groups. The oldest (80–85 years) participants displayed a 50% (p < 0.001) lower activity level compared to the youngest (65–70 years). When using the data to simulate a longitudinal trend, the regression analysis revealed that the decline was equivalent to a rate of 9 cpm (2.8%) per year (B = −9.4, p < 0.001, 95% confidence interval (CI): −7, −12). The oldest age group took on average 5525 steps per day less than the youngest age group (p = 0.02, 95% CI: 626 to 10426), a relative difference of 59%. When using the data to simulate a longitudinal trend, the step variable displayed a yearly decrease of 215 steps (B = −215, p < 0.001, 95% CI: −263, −168).

#### Overall PA level across sex

There were no significant differences in overall physical activity level (cpm) or steps taken per day between women and men within the different age groups (Table 1).

#### Mean minutes per day spent in the different activity categories

Table 2 presents the mean minutes the participants spent in the different activity categories per day. In the two youngest age groups, men spent more time being sedentary compared to women (558 vs. 535 min (p = 0.02) and 554 vs. 525 min (p = 0.03), respectively). Women in all age groups, except for the oldest, spent more minutes in low-intensity PA compared to men (223 vs. 192 min (p < 0.001), 223 vs. 187 min (p < 0.001) and 200 vs. 179 min (p = 0.05), for the 65–69, 70–74, 75–80 year age groups, respectively. No significant sex differences were found within age group when looking at the time spent in lifestyle activities. There was a decline in the proportion of time spent in MVPA when comparing the youngest age group with the oldest (34 vs. 9 min, p < 0.001). A difference between the sexes was only apparent in the 75-79-yr age group where men spent significantly more time in MVPA compared with women. Of the waking hours per day, the whole sample spent 9.3 hours (66%) being sedentary, 3.3 hours (24%) in low-intensity PA, 1 hour (7%) in lifestyle PA, and 30 minutes (3%) in MVPA.

#### Adherence to the physical activity recommendations

A total of 21% of the participants fulfilled the current Norwegian PA recommendations of 30 minutes of daily moderate physical activity, accumulated in bouts of 10 minutes or more (Table 4). The adherence to the recommendations decreased markedly with increasing age and among the 80–85 year-olds 6% adhered to the recommendations. A difference between the sexes were only observed in the 75-79-yr group where men had a significant higher adherence to physical activity recommendations than women (p = 0.01).

**Table 4:** Percentage of the population meeting current PA recommendations

	**Women**	**Men**	**All**
**≥30 min of daily MVPA, in bouts of 10 min or more**			
Age			
65–69 yr	25.0	29.0	27.9^b,c^
70–74 yr	20.3	19.5	19.9
75–79 yr	5.8	22.8^a^	14.8^d^
80–85 yr	7.1	3.0	5.6^e^

^*a*^*p = 0.01 for sex within age group.*

^*b*^*65-69 yr compared to 75–79 yr p = 0.02.*

^*c*^*65-69 yr compared to 80–85 yr p = 0.000.*

^*d*^*75-79 yr compared to 65–69 yr p = 0.02.*

^*e*^*80-85 yr compared to 65–69 yr p = 0.000.*

### Overall PA levels and self-reported health

Physical activity levels differed across categories of self-reported health (Figure 1). Those reporting “very good health” had a 51% higher cpm compared to those in the “poor/very poor health” category (344 (13) vs. 170 (33) cpm, respectively (p < 0.001)), and those reporting to have “good health” had a 43.3% higher cpm compared to those reporting “poor/very poor health” (300 (8) vs. 170 (33) cpm, respectively (p = 0.001)).

## Discussion

The main findings of the present study were that objectively-measured physical activity level significantly differed by age in a national sample of older adults. There were no sex differences in physical activity level within each age group. In the age groups 65–69 years and 70–74 years, men had higher levels of sedentary minutes than women, whilst men in the age group 75–79 years achieved more minutes of MVPA than women. In all age groups, except for the oldest one, women spent significantly more minutes of low-intensity PA than men. Also, overall physical activity was associated with self-reported health.

We found that accelerometer-determined physical activity significantly differed between the different age groups, with the oldest age group having substantially lower mean physical activity levels than the youngest age group. This is in accordance with other cross-sectional studies using the same objective method [10-17]. Our population appeared to have somewhat higher overall physical activity level than what has been reported in other studies [12,16]. While Norwegian men and women in age group 75–79 years had a mean cpm of 256 and 215, respectively, data from this age group in Iceland showed lower physical activity levels (mean cpm 150 and 139 for men and women, respectively) [16]. Our mean physical activity levels in individuals aged 65–74 years are higher than what has been reported among Americans [12]. However, the activity levels in Norway are similar to what has been reported in Sweden [17]. This might be due to differences in socioeconomic status, cultural differences with respect to retirement age, infrastructure and degree of environmental security among the populations studied.

We did not find significant sex differences in physical activity level within each age group, which is in contrast with similar studies from other countries usually showing a higher mean physical activity level among men than among women [10,11,13-16]. This discrepancy might be connected to cultural differences as described above. Also, the lack of a difference in PA level between sexes in the present study is also in contrast to earlier Norwegian studies using self-reported measures of PA [29]. Women may spend more time doing low and lifestyle intensity activities, such as walking, household chores, and gardening [14]. Subjectively-assessed PA have limited accuracy at capturing activities that are unstructured and of low intensity [4], which have a tendency to be performed more often in older populations and in particular among older women [30-32]. This is supported by the fact that Norwegian women spent more time in low-intensity PA and have less sedentary time compared to their male counterparts.

The participants spent the majority of the day being sedentary (66% of the total wear time), and this was followed by low-intensity PA (24%), lifestyle PA (7.1%) and MVPA (3.0%). These findings are comparable to what has been reported among older adults in Iceland [16], Great Britain [14], and Canada [13]. Resent research has also shown dose–response associations between sitting time and mortality from all causes, independent of leisure time physical activity [33]. The large proportion of sedentary time and increased sitting-time is worrying as it might lead to substantial health problems for older people and as a consequence, reduced quality of life and need for assistance. It is therefore important to develop and initiate interventions where the goal is to increase physical activity levels and reduce sedentary time among older adults. In addition to the PA promotion, physicians should also discourage sitting time for extended periods.

When looking at sex- and age trends, Norwegian women are spending less time being sedentary and more time in low-intensity PA per day compared to men at the same age as mentioned above, while men (75-79-yr age group) accumulate more minutes of MVPA than women. In comparison, older men in the UK performed significantly more minutes of MVPA per day than women (23.1 vs. 13.8 min) [14]. Furthermore, the British older adults had a steep decline in the proportion of active time spent in MVPA with increasing age [14], which is in accordance with our results. Similar patterns are also observed among US older adults [10] and among Canadians aged 20–79 years [13], where MVPA decreased across increasing age [10].

The age group 65–69 years averaged 5525 steps more per day than the individuals in age group 80–85 year (p = 0.02), a relative difference of 59%. This is in accordance to what has been found in two other studies [14,15] including older adults, both using accelerometer to assess PA levels. Davis et al. [14] found that younger participants (70–75 years) averaged significantly more steps per day (5661 steps per day) than participants aged 80+ years (3410 steps per day). Harries et al. [15] also showed that step-count declined steadily with age. In the latter study, however, sex differences in step counts were also reported and men achieved 754 more steps daily than women. This is in contrast to the result of the present study where no sex differences in step counts were reported.

Overall, 21% of the participants (women and men: 18% and 22%, respectively) fulfilled the current Norwegian PA recommendations. Data from the United Kingdom shows a similar prevalence among older men (25.6% met national recommendations), but a lower prevalence among older women (14.2%) [14]. In the oldest age group, we found that only 6% reached the national physical activity recommendations. This is a higher percentage compared with a study conducted in the United Kingdom by Harris et al. [15], showing that only 2.5% of the participants 65 years and older met the PA recommendations. On the other hand, looking at the Icelandic oldest (85 years and older), as much as 25% of the men and 9% of the women fulfilled the recommendations, defined as having at least one ≥10 minutes MVPA boats [16]. However, comparability between the current study and the Iceland study [16] is hampered by the use of different physical activity recommendation criteria and differences in data reduction strategies.

In Norway, mean physical activity level declines by approximately 30% between the ages of 9 and 15 years [34]. A further decline of 30% for women and 35% for men have been observed when going from 15 years into adulthood, followed by a stable level of activity until retirement age [23]. Following retirement to 80–85 years, a further decline of 47% in women and 53% in mean PA level was observed in the present study. The causes for these age-related changes in physical activity level are not fully known, although the overall decline of 50% observed during the age of being 65 years to entering 85 years, might be caused by changes in health status and of course the aging process in itself [35]. The higher mean physical activity level in the youngest age group might also be explained by higher prevalence of participants in this age group reporting part- or full time employment than participants in the oldest age group (23% versus 4%). 23% of the youngest age group still reported the fact to be employed. For example, if their work involves a lot of walking and their physical activity measurement period includes only working days then their measured activity level may be higher compared to someone whose measurement period includes non-working days where they may be less active. This will overall affect their computed average activity levels, and has to be taken into consideration.

In the present study significant differences in the overall level of PA were observed between all self-reported health groups, except between those who perceived their health as “either good or bad” and “poor/very poor health”. One of few available studies mentioned above is targeting community-dwelling people in the U.K. from 65 years and older showed that those with poor health took fewer steps compared to those with better health [15]. This difference (p > 0.05) was not found in the current study (data not shown). The latter study used a different method (Health Survey form England, 1988: questions related to general health, disability, long-standing illness, pain, medication use, chronic disease, falls, and walking aid use) to register self-reported health compared to the this study and therefore, the degree of comparability is rather limited. The associations between physical activity level and perceived health are strong, but due to the study design we cannot determine causality.

The major strength of this study is the use of accelerometers to assess physical activity in a relatively large sample of older adults. The participants showed good compliance with the protocol and few data were lost because of insufficient wearing time or defect monitors. Objectively-measured physical activity in combination with self-reported health in older adults, is rather novel. These variables are often presented separately in other studies [11,14,21], and few studies [15] have objectively measured physical activity levels and its association with multiple health factors (e.g. general health).

We acknowledge some limitations to our study. One limitation is the relatively low participation rate. A drop-out analysis performed via registry linkage showed that the responses varied according to socio-demographic variables [23], which is consistent with other population-based studies conducted in Western countries [36].

Furthermore, there are limitations worth noting when interpreting accelerometry data [11]. Accelerometers do not provide qualitative information on the type of physical activities being performed, and hip-mounted accelerometers underestimate upper body movements and activities such as carrying heavy loads, weight training, swimming, and cycling [11]. Nevertheless, accelerometers are sensitive to ambulatory activities such as walking. The participants reported walking as the most frequently performed activity during the measuring period, which decreases the possibility that physical activity level was underestimated [23]. Walking technique must also be taken into consideration because it can affect the validity of accelerometer counts, especially in older individuals [11]. It seems that some accelerometers can undercount activity in individuals with a non-standard gait, e.g. upper body leaned forward and bended knees during walking, thereby underestimate the activity level in these individuals [37]. Furthermore, when interpreting accelerometer data, there is a possibility that the observed differences in physical activity may simply reflect differences in accelerometer wear time between groups. However, there were no significant differences between sexes and between age groups in minutes of daily accelerometer wear time and the sample were compliant to the accelerometer protocol with a mean wear time of 14.0 hours per day.

In the past, methods based on self-ratings of health have been questioned because of their obvious subjective bias [5,6]. Self-reported height and body mass is therefore considered as a limitation to our study. However, several studies have shown that self-report instruments concluding simple measures of health and self-reported functioning in old persons have acceptable reliability and validity [38,39]. Furthermore, because it is inexpensive and easy to administer and interpret, self-reported health is a practical tool suitable for the clinical environment [40] and has become an important variable to assess the state of health in the older population [20,41].

Our findings help to better understand older peoples’ rate of physical activity and thereby help guide the development of needed physical activity interventions targeted at older adults in Norway. The link between PA and prevention of disease, maintenance of independence and improved quality of life in older adults is supported by strong evidence [2,3], and therefore it is of great importance to maintain PA levels as long as possible. Implementation of PA among community-dwelling older adults should therefore be prioritized in the future, with a special focus on the least physically active and the oldest individuals, especially in those with low levels of self-reported health.

## Conclusion

Physical activity level among older adults living in Norway differ by age, where the oldest (80–85 years) displayed a 50% lower activity level compared to the youngest (65–70 years). No sex differences in overall PA level within each age group were observed. Overall, the older people spent 66% of their time being sedentary, 24% in low-intensity PA, 7% in lifestyle PA, and 3% in MVPA. Women spent more time in low-intensity PA, and less time being sedentary and in MVPA compared to men. Overall, 21% of the participants fulfilled the current Norwegian PA recommendations. In the oldest age group, 6% met the recommendations. Physical activity differed across levels of self-reported health and a 51% higher overall level of physical activity was registered in those with “very good health” compared to those with “poor/very poor health. Overall PA levels were associated with self-reported health.

## Abbreviations

CI: Confidence interval; MVPA: Moderate to vigorous physical activity; PA: Physical activity; SD: Standard deviation; SEM: Standard error of the mean.

## Competing interests

The authors declare that they have no competing interests. The results of the present study do not constitute endorsement by the BMC Public Health.

## Authors’ contributions

SAA contributed to the conception and design of the study. BHH was responsible for the collection of the KAN data in corporations with colleagues at nine other test centers throughout Norway. BHH provided the data for analysis. HLS undertook the data analysis and drafted the manuscript. All authors provided critical insight, and revisions to the manuscript. All authors read and approved the final version of the manuscript submitted for publication.

## Pre-publication history

The pre-publication history for this paper can be accessed here:

http://www.biomedcentral.com/1471-2458/14/284/prepub

## References

[B1] Buchner DM Physical activity and prevention of cardiovascular disease in older adults Clin Geriatr Med 2009 25 4 661 675 10.1016/j.cger.2009.08.002 19944266

[B2] Spirduso WW Cronin DL Exercise dose–response effects on quality of life and independent living in older adults Med Sci Sports Exerc 2001 33 6 598 608 10.1097/00005768-200106001-00028 11427784

[B3] Taylor AH Cable NT Faulkner G Hillsdon M Narici M Van Der Bij AK Physical activity and older adults: a review of health benefits and the effectiveness of interventions J Sports Sci 2004 22 8 703 725 10.1080/02640410410001712421 15370483

[B4] Leenders NY Evaluation of methods to assess physical activity in free-living conditions Med Sci Sports Exerc 2001 33 7 1233 1240 11445774 10.1097/00005768-200107000-00024

[B5] Sallis JF Saelens BE Assessment of physical activity by self-report: status, limitations, and future directions Res Q Exerc Sport 2000 71 2 S1 S14 10925819

[B6] Vanhees L Lefevre J Phillippaerts R Martens M Huygens W Troosters T Beunen G How to assess physical activity? How to assess physical fitness? Eur J Cardiovasc Prev Rehab 2005 12 102 114 10.1097/01.hjr.0000161551.73095.9c 15785295

[B7] Mathie MJ Coster ACF Lovell NH Celler BG Accelerometry: providing an integrated, practical method for long-term, ambulatory monitoring of human movement Physiol Meas 2004 25 2 1 20 10.1088/0967-3334/25/2/R01 15132305

[B8] Prince SA Adamo KB Hamel ME Hardt J Connor Gorber SC Tremblay M A comparison of direct versus self-report measures for assessing physical activity in adults: a systematic review Int J Behav Nutr Phys Act 2008 5 56 79 10.1186/1479-5868-5-56 18990237 PMC2588639

[B9] Corder K van Sluijs EM Invited commentary: comparing physical activity across countries - current strengths and weaknesses Am J Epidemiol 2010 171 10 1065 1068 10.1093/aje/kwq068 20406761 PMC3696728

[B10] Evenson KR Buchner DM Morland KB Objective measurement of physical activity and sedentary behaviour among US adults aged 60 years or older Prev Chronic Dis 2012 9 1101 1109 PMC3277387 22172193

[B11] Hawkins MS Storti KL Richardson CR King WC Strath SJ Holleman RG Kriska AM Objectively measured physical activity of USA adults by sex, age, and racial/ethnic groups: a cross-sectional study Int J Behav Nutr Phys Act 2009 6 31 1 7 19493347 10.1186/1479-5868-6-31 PMC2701914

[B12] Troiano RP Berrigan D Dodd KW Mâsse LC Tilert T Mc Dowell M Physical activity in the United States measured by accelerometer Med Sci Sports Exerc 2008 40 1 181 188 10.1249/mss.0b013e31815a51b3 18091006

[B13] Colley RC Garriguet D Janssen I Craig CL Clarke J Tremblay MS Physical activity of Canadian adults: accelerometer results from the 2007 to 2009 Canadian Health Measures Survey Statistics Canada, Catalogue no. 82-003-XPE, Health Reports 2011 22 1 1 10 21510585

[B14] Davis MG Fox KR Hillsdon M Sharp DJ Coulson JC Thompson JL Objectively measured physical activity in a diverse sample of older urban UK adults Med Sci Sports Exerc 2011 43 4 647 654 10.1249/MSS.0b013e3181f36196 20689449

[B15] Harris TJ Owen CG Victor CR Adams R Cook DG What factors are associated with physical activity in older people, assessed objectively by accelerometry? Br J Sports Med 2009 43 442 450 10.1136/bjsm.2008.048033 18487253

[B16] Arnardottir NY Koster A Van Domelen DR Brychta RJ Caserotti P Eiriksdottir G Sverrisdottir JE Launer LJ Gudnason V Johannsson E Harris TB Chen KY Sveinsson T Objectively measurements of daily physical activity patterns and sedentary behavior in older adults: Age, Gene/Environment Suspectibility - Reykjavik Study Age Aging 2013 42 2 222 229 10.1093/ageing/afs160 PMC3575120 23117467

[B17] Hagströmer M Oja P Sjöström M Physical activity and in-activity in adult population assessed by acceleromtry Med Sci Sports Exerc 2007 39 9 1502 1508 10.1249/mss.0b013e3180a76de5 17805081

[B18] Hagströmer M Troiano RP Sjostrom M Berrigan D Levels and patterns of objectively assessed activity – a comparison between Sweden and the United States Am J Epidemiol 2010 171 10 1055 1064 10.1093/aje/kwq069 20406758

[B19] De Bruin A Picavet HSJ Nossikov A Health interview surveys. Towards international harmonization of methods and instruments WHO Reg Publ Eur Ser 1996 58 1 170 8857196

[B20] Ocampo JM Self-rated health: importance of use in elderly adults Colomb Med 2010 41 275 289

[B21] Blomstedt Y Souares A Niamba L Sie A Weinehall L Sauerborn R Measuring self-reported health in low-income countries: piloting three instruments in semi-rural Burkina Faso Glob Health Action 2012 5 8488 8498 10.3402/gha.v5i0.8488 PMC3404415 22833712

[B22] Hamer M Venuraju SM Lahiri A Rossi A Steptoe A Objectively assessed physical activity, sedentary time, and coronary artery calcification in healthy older adults Arterioscler Thromb Vasc Biol 2012 32 2 500 505 10.1161/ATVBAHA.111.236877 22075247

[B23] Hansen BH Kolle E Dyrstad SM Holme I Anderssen SA Accelerometer-determined physical activity in adults and older people Med Sci Sports Exerc 2012 44 2 266 272 10.1249/MSS.0b013e31822cb354 21796052

[B24] Becker W Pedersen A Lyhne N Lyhne N Pedersen JI Pedersen AN Aro A Fogelholm M Torsdottir I Alexander J Anderssen SA Meltzer HM Nordic Nutrition 2004 Recommendations. Integrating nutrition and physical activity 4th ed 2004 Copenhagen (Denmark): Nordic Council of Ministers 1 436

[B25] Healy GN Dunstan DW Salmon J Cerin E Shaw JE Zimmet PZ Owen N Objectively measured light-intensity physical activity is independently associated with 2-h plasma glucose Diabetes Care 2007 30 6 1384 1389 10.2337/dc07-0114 17473059

[B26] Matthews CE Chen KY Freedson PS Buchowski MS Beech BM Pate RR Troiano RP Amount of time spent in sedentary behaviors in the United States, 2003–2004 Am J Epidemiol 2008 167 7 875 881 10.1093/aje/kwm390 18303006 PMC3527832

[B27] Matthews CE Calibration of accelerometer output for adults Med Sci Sports Exerc 2005 37 11 512 522 10.1249/01.mss.0000185659.11982.3d 16294114

[B28] Trost SG McIver KL Pate RR Conducting accelerometer-based activity assessments in field-based research Med Sci Sports Exerc 2005 37 11 531 543 10.1249/01.mss.0000185657.86065.98 16294116

[B29] Anderssen SA Engeland A Søgaard AJ Nystad W Graff-Iversen S Holme I Changes in physical activity behavior and the development of body mass index during the last 30 years in Norway Scand J Med Sci Sports 2008 18 3 309 317 10.1111/j.1600-0838.2007.00645.x 17645730

[B30] Ainsworth BE Evaluation of the Kaiser physical activity survey in women Med Sci Sports Exerc 2000 32 7 1327 1338 10.1097/00005768-200007000-00022 10912901

[B31] King AC Rejeski WJ Buchner DM Physical activity interventions targeting older adults. A critical review and recommendations Am J Prev Med 1998 15 4 316 333 10.1016/S0749-3797(98)00085-3 9838975

[B32] King AC Interventions to promote physical activity by older adults J Gerontol A Biol Sci Med Sci 2001 56 2 36 46 11730236 10.1093/gerona/56.suppl_2.36

[B33] Katzmarzyk PT Church TS Craig CL Bouchard C Sitting time and mortality from all causes, cardiovascular disease, and cancer Med Sci Sports Exerc 2009 41 5 998 1005 10.1249/MSS.0b013e3181930355 19346988

[B34] Kolle E Steene-Johannessen J Andersen LB Andersen SA Objectively assessed physical activity and aerobic fitness in a population-based sample of Norwegian 9- and 15-year-olds Scand J Med Sci Sports 2010 20 1 41 47 10.1111/j.1600-0838.2009.00892.x 19422647

[B35] Kokkinos P Physical activity, health benefits, and mortality risk Cardiology 2012 1 14 10.5402/2012/718789 PMC3501820 23198160

[B36] Sogaard AJ Selmer R Bjertness E Thelle D The Oslo health study: the impact of self-selection in a large, population-based survey Int Equity Health 2004 3 1 3 10.1186/1475-9276-3-3 PMC428581 15128460

[B37] Storti KL Peetee KK Brach JS Talkowski JB Richardson CR Kriska AM Gait speed and step-count monitor accuracy in community-dwelling older adults Med Sci Sports Exerc 2008 40 1 59 64 10.1249/mss.0b013e318158b504 18091020

[B38] O’Brien Cousins S Validity and reliability of self-reported health of persons aged 70 and older Health Care Women Int 1997 18 2 165 174 10.1080/07399339709516271 9119792

[B39] Siu AL Hays RD Ouslander JG Osterwell D Valdez RB Krynski M Gross A Measuring functioning and health in very old J Geront Med Sci 1993 48 10 14 10.1093/geronj/48.1.M10 8418139

[B40] DeSalvo KB Fisher WP Tran K Bloser N Merill W Peabody J Assessing measurement properties of two single-item general health measures Qual Life Res 2006 15 191 201 10.1007/s11136-005-0887-2 16468076

[B41] Lundberg O Manderbacka K Assessing reliability of a measure of self-rated health Scan J Soc Med 1996 24 218 224 10.1177/140349489602400314 8878376

